# Systematic Review of Abstinence-Plus HIV Prevention Programs in High-Income Countries

**DOI:** 10.1371/journal.pmed.0040275

**Published:** 2007-09-18

**Authors:** Kristen Underhill, Don Operario, Paul Montgomery

**Affiliations:** Centre for Evidence-Based Intervention, University of Oxford, Oxford, United Kingdom; University of California Los Angeles, United States of America

## Abstract

**Background:**

Abstinence-plus (comprehensive) interventions promote sexual abstinence as the best means of preventing HIV, but also encourage condom use and other safer-sex practices. Some critics of abstinence-plus programs have suggested that promoting safer sex along with abstinence may undermine abstinence messages or confuse program participants; conversely, others have suggested that promoting abstinence might undermine safer-sex messages. We conducted a systematic review to investigate the effectiveness of abstinence-plus interventions for HIV prevention among any participants in high-income countries as defined by the World Bank.

**Methods and Findings:**

Cochrane Collaboration systematic review methods were used. We included randomized and quasi-randomized controlled trials of abstinence-plus programs for HIV prevention among any participants in any high-income country; trials were included if they reported behavioural or biological outcomes. We searched 30 electronic databases without linguistic or geographical restrictions to February 2007, in addition to contacting experts, hand-searching conference abstracts, and cross-referencing papers. After screening 20,070 abstracts and 325 full published and unpublished papers, we included 39 trials that included approximately 37,724 North American youth. Programs were based in schools (10), community facilities (24), both schools and community facilities (2), health care facilities (2), and family homes (1). Control groups varied. All outcomes were self-reported. Quantitative synthesis was not possible because of heterogeneity across trials in programs and evaluation designs. Results suggested that many abstinence-plus programs can reduce HIV risk as indicated by self-reported sexual behaviours. Of 39 trials, 23 found a protective program effect on at least one sexual behaviour, including abstinence, condom use, and unprotected sex (baseline *n* = 19,819). No trial found adverse program effects on any behavioural outcome, including incidence of sex, frequency of sex, sexual initiation, or condom use. This suggests that abstinence-plus approaches do not undermine program messages encouraging abstinence, nor do they undermine program messages encouraging safer sex. Findings consistently favoured abstinence-plus programs over controls for HIV knowledge outcomes, suggesting that abstinence-plus programs do not confuse participants. Results for biological outcomes were limited by floor effects. Three trials assessed self-reported diagnosis or treatment of sexually transmitted infection; none found significant effects. Limited evidence from seven evaluations suggested that some abstinence-plus programs can reduce pregnancy incidence. No trial observed an adverse biological program effect.

**Conclusions:**

Many abstinence-plus programs appear to reduce short-term and long-term HIV risk behaviour among youth in high-income countries. Programs did not cause harm. Although generalisability may be somewhat limited to North American adolescents, these findings have critical implications for abstinence-based HIV prevention policies. Suggestions are provided for improving the conduct and reporting of trials of abstinence-plus and other behavioural interventions to prevent HIV.

## Abstract 2

In their systematic review, Underhill and colleagues found that abstinence-plus programs appear to reduce short-term and long-term HIV risk behavior among youth in high-income countries.

## Editors' Summary

**Background.:**

Human immunodeficiency virus (HIV), which causes AIDS, is most often spread through unprotected sex (vaginal, oral, or anal) with an infected partner. Individuals can reduce their risk of becoming infected with HIV by abstaining from sex or delaying first sex, by being faithful to one partner or having few partners, and by always using a male or female condom. Various HIV prevention programs targeted at young people encourage these protective sexual behaviors. Abstinence-only programs (for example, Project Reality in the US) present no sex before marriage as the only means of reducing the risk of catching HIV. Abstinence-plus programs (for example, the UK Apause program) also promote sexual abstinence as the safest behavior choice to prevent HIV infection. However, recognizing that not everyone will remain abstinent, and that in many locations same-sex couples are not permitted to marry, abstinence-plus programs also encourage young people who do become sexually active to use condoms and other safer-sex strategies. Safer-sex programs, a third approach, teach people how to protect themselves from pregnancy and infections and might recommend delaying first sex until they are physically and emotionally ready, but do not promote sexual abstinence over safer-sex strategies such as condom use.

**Why Was This Study Done?:**

There is considerable controversy, particularly in the US, about the relative merits of abstinence-based programs for HIV prevention. Abstinence-only programs, which the US government supports, have been criticized because they provide no information to protect participants who do become sexually active. Critics of abstinence-plus programs contend that teaching young people about safer sex undermines the abstinence message, confuses participants, and may encourage them to become sexually active. Conversely, some people worry that the promotion of abstinence might undermine the safer-sex messages of abstinence-plus programs. Little has been done, however, to look methodically at how these programs change sexual behavior. In this study, the researchers have systematically reviewed studies of abstinence-plus interventions for HIV prevention in high-income countries to get an idea of their effect on sexual behavior.

**What Did the Researchers Do and Find?:**

In an extensive search for existing abstinence-plus studies, the researchers identified 39 trials done in high-income countries that compared the effects on sexual behavior of various abstinence-plus programs with the effects of no intervention or of other interventions designed to prevent HIV infection. All the trials met strict preset criteria (for example, trial participants had to have an unknown or negative HIV status), and all studies meeting the criteria turned out to involve young people in the US, Canada, or the Bahamas, nearly 40,000 participants in total. In 23 of the trials, the abstinence-plus program studied was found to improve at least one self-reported protective sexual behavior (for example, it increased abstinence or condom use) when compared to the other interventions in the trial; none of the trials reported a significant negative effect on any behavioral outcome. Limited evidence from a few trials indicated that some abstinence-plus programs reduced pregnancy rates, providing a biological indicator of program effectiveness. Conversely, there were no indications of adverse biological outcomes such as an increased occurrence of sexually transmitted diseases in any of the trials.

**What Do These Findings Mean?:**

These findings indicate that some abstinence-plus programs reduce HIV risk behavior among young people in North America. Importantly, the findings do not uncover evidence of any abstinence-plus program causing harm. That is, fears that these programs might encourage young people to become sexually active earlier or confuse them about the use of condoms for HIV prevention seem unfounded. These findings may not apply to all abstinence-plus programs in high-income countries, do not include low-income countries, do not specifically address nonheterosexual risk behavior, and are subject to limited reliability in self-reporting of sexual activity by young people. Nonetheless, this analysis provides support for the use of abstinence-plus programs, particularly in light of another systematic review by the same authors (A systematic review of abstinence-only programs for prevention of HIV infection, published in the *British Medical Journal*), which found that abstinence-only programs did not reduce pregnancy, sexually transmitted diseases, or sexual behaviors that increase HIV risk. Abstinence-plus programs, these findings suggest, represent a reasonable strategy for HIV prevention among young people in high-income countries.

**Additional Information.:**

Please access these Web sites via the online version of this summary at http://dx.doi.org/10.1371/journal.pmed.0040275.

• US National Institute of Allergy and Infectious Diseases fact sheet on HIV infection and AIDS

• Information from the UK charity AVERT on all aspects of HIV and AIDS, including HIV and AIDS prevention

• US Centers for Disease Control and Prevention fact sheet on HIV/AIDS among young people (in English and Spanish)

• Information on Project Reality, a US abstinence-only program

• Information on Reducing the Risk and on Apause, US and UK abstinence-plus programs, respectively

## Introduction

Although the HIV epidemic is most devastating in middle- and low-income countries, new infections continue multiplying even in countries with many resources for prevention [1]. The World Health Organization estimated in 2004 that 1.6 million people in high-income countries were living with HIV [2]; by 2006, approximately 2.1 million individuals in North America, Western Europe, and Central Europe were HIV-positive [3]. Sexual behaviour is the most common transmission route in high-income countries, and primary prevention efforts remain crucial among high-risk groups and the general population.

Abstinence-plus programs are popular strategies for HIV prevention in some high-income countries, particularly in the United States. These interventions promote sexual abstinence as the safest behaviour choice to prevent HIV; however, recognizing that some participants are not abstinent, they also encourage sexually active participants to use condoms and other safer-sex strategies. Also known as “comprehensive” and “abstinence-oriented” approaches, abstinence-plus programs focus primarily on individual-level determinants of risk behaviour. Typical program components include communication or condom skills practice and HIV education. Abstinence-plus programs primarily target young people and are delivered via schools, community-based organizations, clinics, and media-based campaigns.

Abstinence-plus programs differ from abstinence-only approaches, which present abstinence as the exclusive means of risk reduction (without encouraging condom use or other prevention strategies). Both program types differ from safer-sex programs, which do not prioritize abstinence over condom use. Furthermore, both strategies are controversial. Abstinence-only interventions have been criticized for omitting condom promotion [4–9], while critics of abstinence-plus programs have suggested that safer-sex promotion can “undermine the abstinence message” [10], confuse program participants, or encourage sex [11]. Conversely, it is also important to investigate whether the promotion of abstinence can undermine the safer-sex messages of abstinence-plus programs [12].

This review focuses on studies conducted in high-income countries. A previous systematic review of abstinence-based programs in low-income countries has shown minimal to no effects on behavioural outcomes [13]. No internationally focused, systematic, and HIV-specific review of abstinence-plus programs has been conducted in high-income countries. Evidence from the developing world may not apply to more affluent countries because of epidemiological, structural, and methodological differences [14,15]. Abstinence-plus programs might be implemented or evaluated differently in high-income economies, particularly given differences in risk groups, resource availability, and HIV prevalence. We focused on high-income countries as a means of limiting heterogeneity and producing more specific results.

The scope and methodology of this review are unique. Prior reviews in high-income countries have examined abstinence-plus interventions alongside abstinence-only interventions [16,17], alongside safer-sex programs [18–20], or in analyses of all three program types [12,21–33]. These strategies can make it difficult to determine which programs used an abstinence-plus hierarchy. Four reviews have suggested that abstinence-plus programs can reduce sexual risks [17,34–36], but these reviews are limited: all four were limited to US and Canadian adolescents, three focused on pregnancy instead of HIV [17,34,35], three included quasi-experimental program evaluations [34–36], and none included evidence from trials completed since 2004.

Evidence for abstinence-plus programs has immediate implications for public health in high-income countries, which encompass marked inequalities in income, health, and HIV prevalence [37]. The subgroups at highest risk encounter a number of structural and social risk factors [38], such as discrimination and poverty [39,40]. In the United States, for example, HIV infection is concentrated among African American and Hispanic minorities, young people, low-income urban residents, and rural Southern populations [1]. Our review was designed to identify studies among both privileged and underprivileged groups in high-income countries.

This review sought to identify, appraise, and synthesize evaluations of abstinence-plus interventions for HIV prevention in high-income countries. This version complements our full Cochrane systematic review [41,42] and our prior study of abstinence-only interventions for HIV prevention in high-income countries [43,44], which found that abstinence-only programs neither decrease nor exacerbate HIV risk in high-income settings.

## Methods

### Eligibility Criteria

Eligibility criteria are listed in Box 1 and further clarified in this section.

**Box 1. Summary of Eligibility Criteria**

**Inclusion criteria**

1. Randomized or quasi-randomized controlled trial

2. Trial took place in a high-income economy (defined by the World Bank)

3. Participants were of unknown or negative HIV status

4. At least one trial arm received an abstinence-plus intervention (i.e., an intervention that emphasized sexual abstinence as the most effective means of HIV prevention, but also actively promoted safer sex)

5. HIV prevention was a stated program goal

6. Trial reported a biological or behavioural outcome

**Exclusion criteria**

1. Trial did not use a randomized or quasi-randomized controlled design

2. Trial did not take place in a high-income economy

3. All participants were known to be HIV-positive

4. No trial arm received an abstinence-plus intervention

5. HIV prevention was not a program goal

6. Trial did not report a biological or behavioural outcome

#### Inclusion criteria.

We included randomized and quasi-randomized controlled trials. Quasi-randomized controlled trials approximate randomization by using a method of allocation that is unlikely to lead to consistent bias, such as alternating participants. We made no exclusions by type of control group. We included trials that took place among any participants of negative or unknown serostatus in any high-income economy. High-income economies are defined by the World Bank as those with a gross national income per capita of US$10,726 or higher [45], listed in Text S1.

Interventions were any efforts that encouraged sexual abstinence as the most effective means of HIV prevention, but also promoted safer-sex strategies such as condom use or partner reduction. Although definitions of terms such as “abstinence” and “sex” vary and are often not specified [46–51], trials were included if programs encouraged participants to reduce, delay, or stop sexual activity. Sexual activity could mean vaginal sex, oral sex, anal sex, or any combination thereof. We understood “abstinence” to mean refraining from sexual activity whether it is protected or unprotected. Trials were included if HIV prevention was a stated program goal. Upon discussion with the Cochrane HIV/AIDS Group, we included trials of programs that aimed to prevent both pregnancy and HIV, as well as trials of programs that aimed only to prevent HIV. We made no exclusions by the type of organization or facilitators delivering the program.

We extracted outcome data for biological outcomes (e.g., HIV incidence), behavioural outcomes (e.g., unprotected vaginal sex), and HIV/AIDS-related knowledge. We included same-sex sexual behaviour outcomes.

#### Exclusion criteria.

Because we were interested in primary prevention of HIV infection, trials limited to HIV-positive participants were excluded. No exclusions were made by any other participant characteristic within high-income countries, including age.

We excluded trials that focused exclusively on pregnancy prevention without listing HIV prevention as a program goal. Programs that focus only on pregnancy may neglect the HIV-related risks of oral sex, anal sex, same-sex sexual activity, and nonsexual means of transmission. Including trials of these programs might have augmented statistical heterogeneity or masked the effects of programs that did aim to prevent HIV.

Trials that did not report a biological or behavioural outcome were excluded; although knowledge, intentions, and attitudes can mediate intervention effects [27,52], they may be less reliable indicators of behaviour or HIV risk [53–58].

### Information Sources

Our search was designed to identify published and unpublished program trials. We searched 30 electronic databases from January 1980 to February 2007 (Box 2). Electronic searches were not restricted by country, geography, economic characteristics, participant group, outcome measure, or language of publication. We also searched libraries of international agencies involved with HIV prevention (e.g., UNAIDS, WHO, CDC) and hand-searched relevant conference abstracts from 2000 onward (i.e., International AIDS Conference, meeting of the International Society for Sexually Transmitted Diseases Research, Conference on Retroviruses and Opportunistic Infections, the Abstinence Education Evaluation Conference, and the US National HIV Prevention Conference). We searched for unpublished and ongoing research by contacting field experts and cross-referencing papers.

**Box 2. Electronic Databases Searched**

ADOLEC, AIDSLINE, AMED, ASSIA, BiblioMap, BIOSIS, BNI, Catalog of US Government Publications, CHID, CINAHL, CENTRAL (Cochrane Central Register of Controlled Trials), DARE, Dissertation Abstracts International, EMBASE, ERIC, EurasiaHealth Knowledge Multilingual Library, Global Health Abstracts, HealthPromis, HMIC, PAIS, Political Science Abstracts, PsycINFO, PubMed, RCN, SCISEARCH, SERFILE, SIGLE, Social Services Abstracts, Sociological Abstracts, TRoPHI

### Search

We developed our search strategy in consultation with the Cochrane HIV/AIDS Group and additional trial search experts. We included terms specific to HIV, abstinence-based interventions, and comparative study designs. Our PubMed search is included in Text S2 and was modified as needed for other databases; database-specific search strategies appear in the forthcoming Cochrane review and are available from the authors.

Our search was designed to identify program trials measuring any biological, behavioural, cognitive, attitudinal, or other outcome; we excluded trials without behavioural or biological outcomes only after examining full reports. To heighten sensitivity, our search was designed to identify trials of both abstinence-only and abstinence-plus programs. We reviewed full program descriptions before excluding trials of abstinence-only programs and trials of abstinence-plus programs that did not aim to prevent HIV.

### Data Collection

Two reviewers independently assessed all abstracts for inclusion, resolving disagreements by discussion and referral to the third reviewer. Reviewers were not blinded to any aspect of the trials. If any reviewer believed a record to be relevant, a full-text copy was retrieved. Trialists were contacted for clarification as needed.

Using standard forms, two reviewers independently extracted data and assessed evaluations for methodological quality. Where several reports of a single evaluation existed, data were extracted from all available reports. Disagreements were resolved by discussion and referral to the third reviewer. Multiple attempts were made to contact trialists for missing data, but nonresponse and data loss by trialists were common.

### Assessment of Trial Quality

We assessed methodological quality following the Cochrane Handbook [59], and we highlighted attrition as a particular limitation of any trials with a total dropout exceeding 33% of baseline enrolment. Where available, we also assessed information about cost, acceptability, and implementation (i.e., program design, actual delivery by clinicians, actual uptake by participants, and context [60]). Additional details of our methods are provided in the Cochrane review [41,42].

### Analysis and Presentation of Results

All trials were summarized in Review Manager (RevMan) version 4.2 (Cochrane Information Management System, http://www.cc-ims.net/RevMan) where possible. We determined that quantitative synthesis was inappropriate because of data unavailability, lack of intention-to-treat analyses, and heterogeneity in programs, clinical settings, control groups, outcome measures, and evaluation designs. Individual trial results are presented as derived from RevMan. Where we were unable to reanalyze data, we report results from the primary trials. When our reanalysis differed from published trial reports (specified in the discussion), we report the reanalyzed results. Data limitations made it impossible to statistically test for publication bias.

When trials used cluster randomization, we followed procedures outlined by the Cochrane Handbook [59] and Johnson et al. [61] to adjust for intraclass correlation. Three evaluations reported trial-specific intraclass correlation coefficients, which we used in our analyses for those trials [62–64]. We were unable to obtain trial-specific intraclass correlation coefficients or raw data for the remaining cluster-randomized trials; we therefore followed the precedent of using intraclass correlation coefficients of 0.015 for school-based evaluations and 0.005 for community-based evaluations [61].

### Ethical Approval

This review dealt entirely with secondary sources. Ethical approval was secured through the Department of Social Policy and Social Work, University of Oxford.

## Results

**Figure 1:**
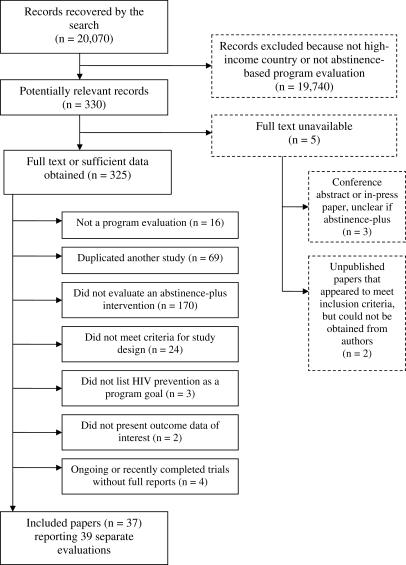
Flow Chart of Search Results

The search retrieved 20,070 records (i.e., citations and abstracts), of which 330 were deemed potentially relevant evaluations by any reviewer (Figure 1). We successfully obtained full versions of 325 reports. After excluding reports based on study design, intervention description, and outcomes of interest, we included 39 trials [62–100] from 37 separate primary papers. Seven trials were unpublished [71,73,76,80,84,89,100], and conference presentations constituted the primary source of information for three evaluations [71,80,84]. We included conference presentations when we were able to obtain missing data from authors or when there was no evidence of selective reporting of results. Authors were contacted on multiple occasions for missing information. Data were also extracted from supplementary papers where possible (Danella, et al. unpublished data and [101–122]).

### Description of Studies

Included studies are described in Table S1 and Table 1.

**Table 1:**
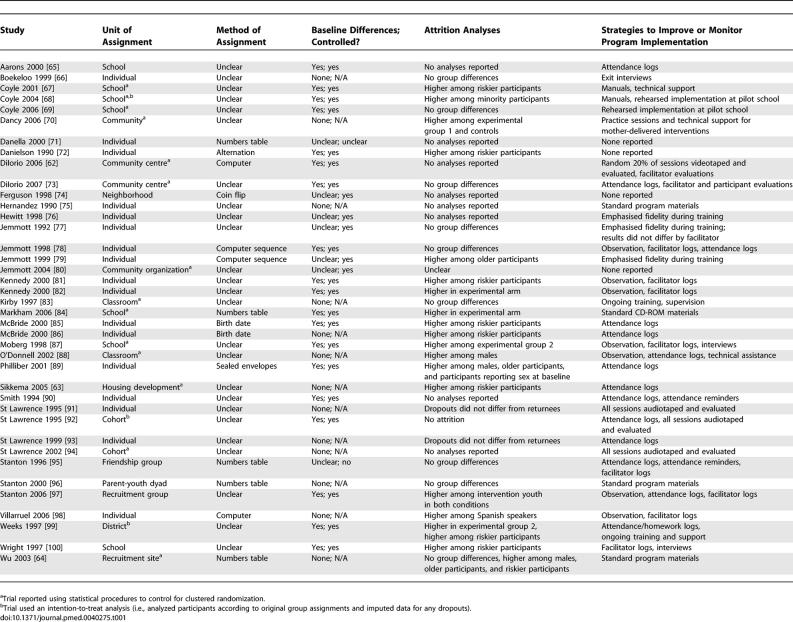
Study Design

#### Participants.

Despite our international search, all 39 trials included youth only from the US, Canada, and the Bahamas. Together, the trials enrolled approximately 37,724 participants at baseline (median enrolment = 535, totals were approximated for several studies with nonspecific reporting). Mean participant ages for individual trials ranged from 11.3 [95] to 19.3 y [75], with a median of 14.0 y across trials. Twenty-nine trials enrolled primarily minority participants [62–70,73,74,76–84,88–91,93,95,96,98,99], and 18 indicated that participants were of lower socioeconomic status than the general population [62–65,70,73,74,77–79,84,88–91,95,96,99].

#### Interventions.

Every intervention promoted sexual abstinence and condom use for HIV prevention, presenting abstinence as the most effective choice. Specific behavioural and temporal definitions of “abstinence” (e.g., “refraining from oral, anal, and vaginal intercourse until marriage”) were rarely provided. The majority of programs were school-based (ten programs [65,67–69,75,83,84,87,99,100]) or community-based (24 programs [62–64,70,71,73,74,76–82,85,86,89–95,98]), and all but three interventions [66,72,96] took place in group settings. Pregnancy prevention was emphasised along with HIV prevention in 15 programs [62,65,67–69,72,74,83–90]; the remaining programs targeted HIV only. Exposure ranged from 30 min [66] to 2,250 program hours over 3 y [89] (median exposure = approximately 10.5 h). Program facilitators were most commonly adults (29 programs [62,64,66,68–73,76,77,79,80,82,84–99]) or both adults and peers (six programs [63,65,67,74,78,100]).

Several programs were represented in multiple evaluations. These included Be Proud! Be Responsible! (eight trials [76–82,98]), Becoming a Responsible Teen (four trials [91–94]), Focus on Kids (four trials [64,71,95,97]), and the ImPACT parental monitoring program (two trials [64,96]).

#### Study design.

Under-reporting hindered assessment of methodological quality. Five studies were quasi-randomized controlled trials [72,74,85–87]; all other trials reported using random assignment, but only ten reported the method of generating the allocation sequence [62,64,71,78,79,84,89,95,96,98]. No systematic differences were observed between the results of quasi-randomized and randomized controlled trials. Clusters of participants (e.g., schools) were randomized in 21 trials [62–65,67–70,73,74,80,83,84,87,88,92,94,95,97,99,100], of which 14 reported analyses that accommodated the blocked unit of randomization [62–64,67–70,73,80,83,84,87,88,94].

Attrition at final follow-up ranged from 0% [92] to 58% [69], with a median final attrition of 20% of baseline enrolment. Final attrition exceeded 33% in eight trials [63,64,68,69,81,82,99,100]. Almost every trial analyzed participants in their original arms without imputing data for dropouts (i.e., complete case analyses [123]), making results vulnerable to attrition bias. There were five exceptions: one analyzed participants by program exposure [88], three had no attrition or used intention-to-treat procedures that imputed data for dropouts [68,92,99], and analytic procedures for one were unclear [80].

Control groups varied as described in Table S1. The use of “usual care” controls was problematic, as it was often unclear what services these groups actually received. In 15 trials, several treatment arms that each received a different abstinence-plus intervention were included [62–64,70,74,76,78,87,88,91,92,94,95,97,99]; in most of these, one arm was enhanced by an extra component (e.g., community service or skills training). Many trials used an attention control group, which consisted of a program that was equal in format and duration to the experimental program, but was not an abstinence-plus intervention. Only one trial explicitly compared an abstinence-plus program against an abstinence-only and a safer-sex intervention [75].

#### Outcome measures.

All outcomes were self-reported. Eight trials used computers or audio computer assisted self-interviewing (ACASI) technology to gather data [63,64,70,73,84,95–97], one trial collected outcome data by phone [66], and the remaining trials used written surveys or did not specify methods of data collection. Limitations of self-reported data have been analyzed elsewhere with respect to adolescent sexual behaviour [55,124–135]. Recent investigations (with exceptions [136]) have suggested that ACASI technology minimizes self-report bias among adolescents [137–139], but there were too few ACASI trials in this review to observe differences in results by data collection method.

**Figure 2:**
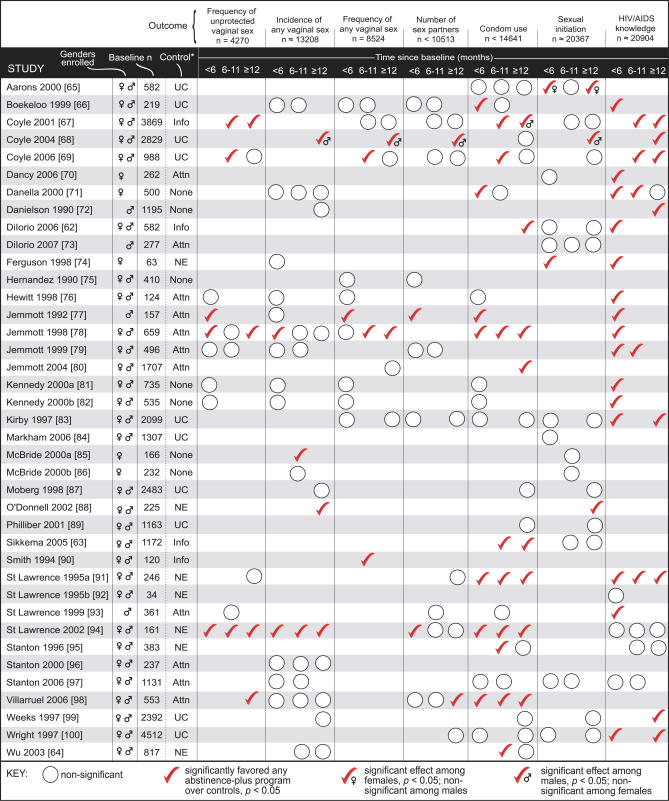
Trials Reporting Behavioural Outcomes and HIV/AIDS Knowledge For each outcome *n* refers to the total number of participants analyzed at any follow-up. Where no symbol appears, the outcome was either not measured or not reported. No harms were observed. All results were reanalyzed in RevMan software where possible, controlling for clustering. If two or more data points fell into the same follow-up range (e.g., 12 mo and 24 mo assessments), a significant effect at any follow-up is reported. If an outcome was measured more than one way (e.g., percentage of condom-protected intercourse occasions, condom use at last intercourse), a significant effect for any definition is reported. *Attn, attention-matched program that did not focus on HIV prevention; Info, information about HIV; NE, “nonenhanced” program version; None, no treatment; UC, usual care.

As presented in Table 2 and Figure 2, we classified follow-up assessments as short-term (<6 mo after baseline), medium-term (6–11 mo), and long-term (≥12 mo). Median final assessment time was 12 mo after baseline. Follow-up assessments took place at a wide variety of times after interventions ended, and trials varied in the recall periods over which sexual behaviours were assessed (e.g., over the last 3 mo, ever). Twenty trials could be partially or completely reanalyzed in RevMan [62–64,66,70,74,77–79,83,85–87,89,90,92,95,97–99].

**Table 2:**
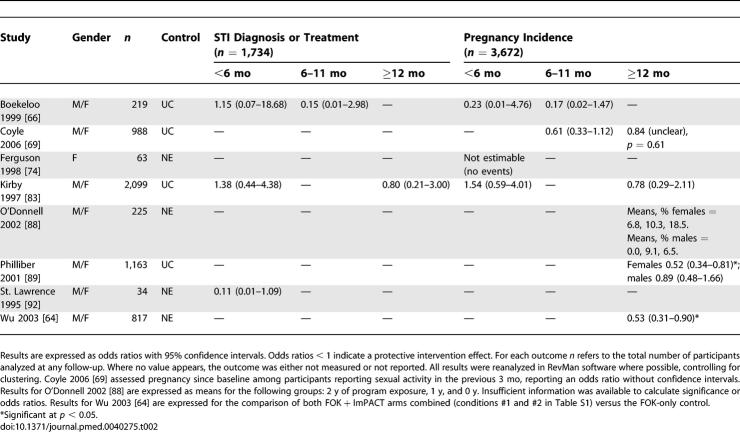
Trials Reporting Biological Outcomes

The outcomes of greatest relevance to HIV risk (i.e., HIV infection, sexually transmitted infection [STI], and unprotected sexual behaviours) were underutilized. No trial assessed HIV incidence. Seven trials assessed self-reported STI or pregnancy [64,66,69,74,83,88,89], which are vulnerable to floor effects (i.e., incidence rates are so low that trials may be underpowered to find significant differences between groups) and underreporting due to unknown status. An additional trial used records to assess the receipt of STI treatment [92].

Every trial reported at least one behavioural outcome. Nine trials assessed oral or anal sex acts [64,66,70,77,79,84,91,93,100], but three reported these only in summary measures of oral, anal, and vaginal intercourse [66,70,84]. Only three trials reported specific definitions of “sex” (e.g., “a boy's penis in your vagina/your penis in a girl's vagina” [76]), all of which signified vaginal intercourse [68,76,98]. Given this trend, we classified results for nonspecific definitions of “sex” as vaginal sex. No trial reported same-sex sexual behaviour outcomes.

### Biological Outcomes

Table 2 presents odds ratios and 95% confidence intervals, where calculable, for biological outcomes. No trial observed a significantly adverse biological effect. In these results, *n* refers to the number of participants included in the analyses and is not necessarily equivalent to the number enrolled at baseline or retained at follow-up. Some totals are approximated due to nonspecific reporting in primary trials.

Two trials measured self-reported STI diagnosis by a doctor or nurse [66,83], reporting data for 1,700 participants at any follow-up. Neither found significantly protective intervention effects compared to usual care, although nonsignificant differences favoured the abstinence-plus programs. A similarly nonsignificant difference was observed in a third trial (*n* = 34), which assessed participants' receipt of STI treatment [92].

Limited evidence suggested that abstinence-plus programs can reduce pregnancy incidence. Seven trials assessed self-reports of having or causing a pregnancy, with analyses representing 3,672 participants [64,66,69,74,83,88,89]. One trial discovered a significantly protective long-term effect compared to usual care (*n* = 941), although subgroup analyses found significance among girls only (*n* = 519) [89]. Another found a significantly protective effect compared to a nonenhanced program version (*n* = 494) [64]. A third trial did not report significance, but long-term findings suggested that students who participated in a school-based curriculum with community service were less likely to report pregnancy than students without service involvement (*n* = 195) [88]. The remaining four trials found no significant effects (*n* = 2,053) [66,69,83].

### Behavioural Outcomes

Every trial reported at least one behavioural outcome, and 23 trials found a significantly protective intervention effect on any sexual risk behaviour [62–69,71,73,74,77–80,84,85,88,90,91,94,95,98]. No trial observed an adverse effect on any behavioural outcome. Figure 2 presents each trial's findings on the most commonly reported outcome measures. Less commonly reported outcomes appear in text only.

#### Incidence and frequency of unprotected sex.

Three trials assessed participants' self-reported incidence of unprotected vaginal sex (*n* < 2,495), using attention control groups [73,78,80]. Two trials found significantly protective effects at 12 mo follow-up. One assessed lifetime incidence of unprotected sex among sexually experienced participants (*n* < 277) [73]; the other assessed unprotected sex in the past 3 mo, finding protective effects only among participants who were sexually experienced at baseline (*n* ≈ 69) [78]. The third trial found no significant effect at 12 mo follow-up (*n* ≈ 1,707) [80].

Participants' self-reported frequency of unprotected vaginal sex was assessed in 12 trials (*n* = 4,270) [67,69,76–79,81,82,91,93,94,98]. Six found protective intervention effects compared to attention controls (*n* = 762) [77,78,98], information about HIV (*n* = 1,371) [67], usual care (*n* = 412) [69], or a nonenhanced program version (*n* = 159) [94]. Long-term effects in one trial were significant only among participants who reported sexual experience at baseline (*n* ≈ 69) [78]. The remaining six trials found no significant effects (*n* = 1,353) [76,79,81,82,91,93].

In two trials participants' self-reported frequency of unprotected anal sex was assessed (*n* = 537), and both evaluated versions of the Becoming a Responsible Teen program [91,93]. One took place in a juvenile reformatory and found no significant program effects approximately 7 mo after baseline compared to an attention control (*n* = 312) [93]. The other took place in a community-based organization and found a significantly protective effect over a 14 mo follow-up, compared to a nonenhanced version (*n* = 225) [91]. This was also the only evaluation to assess frequency of unprotected oral sex, discovering a significantly protective long-term effect (*n* = 225).

#### Incidence and frequency of all sex.

Incidence of any (protected or unprotected) vaginal sex was evaluated in 21 trials (*n* ≈ 13,208) [64,66,68,71,72,74,76–79,81,82,85–88,94,96–99]. Significantly protective effects at any time point were found in five trials compared to no treatment (*n* = 55) [85], attention controls (*n* = 356) [78], usual care (among males only, *n* = 1,412) [68], or a nonenhanced program version (*n* = 414) [88,94]; the remaining 16 trials found no significant effects (*n* = 9,379) [64,66,71,72,74,76,77,79,81,82,86,87,96–99].

Frequency of recent vaginal sex was reported in 13 trials (*n* = 8,524) [66–69,75–78,80–83,90]. In five trials significantly protective effects were observed at any time point compared to attention controls (*n* = 169) [77,78], information about HIV (*n* = 95) [90], or usual care (*n* = 1,905) [68,69]. Protective effects in one trial using an attention control were significant only among participants who were sexually experienced at baseline (*n* ≈ 69) [78]. The other eight trials found no significant effects (*n* = 4,371) [66,67,75,76,80–83].

Incidence of anal sex was reported in three trials (*n* = 1,091) [64,77,79]. Two compared versions of Be Proud! Be Responsible! to attention controls, assessing anal sex in the past 3 mo; each found a protective effect at longest follow-up, which was 3 mo in one trial (*n* = 117) [77] and 6 mo in the other (*n* = 460) [79]. These trials also assessed frequency of anal sex; one found a protective effect at 6 mo follow-up (*n* = 460) [79]. The third trial compared three active interventions: the Focus on Kids program with parental monitoring and booster sessions, the program with parental monitoring only, and the program without parental monitoring or boosters [64]. At 24 mo follow-up, no pairwise comparison showed a significant program effect on anal sex in the past 6 mo (*n* = 494).

One trial reported frequency of oral sex, comparing the Becoming a Responsible Teen program to an attention control; findings were nonsignificant approximately 7 mo after baseline (*n* = 312) [93].

In four trials incidence of any recent oral, anal, or vaginal sex was reported (*n* = 5,084) [66,84,91,100]. Two trials found protective effects: one compared to usual care at 5 mo follow-up (*n* = 1,206) [84], and one compared to a nonenhanced program version at 14 mo follow-up (*n* = 225) [91]. The remaining two trials found no significant effects compared to usual care (*n* = 3,653) [66,100].

One trial assessed the incidence of “casual sex” at 3.5 mo follow-up (*n* = 34) [92]; although results favoured the addition of skills training to the information-focused Becoming a Responsible Teen program, findings did not reach significance when analysed in RevMan. Another trial of the same program assessed frequency of casual sex approximately 7 mo after baseline (*n* = 312), finding no significant effects compared to an attention control [93].

#### Number of partners.

In 13 trials participants' number of sexual partners was assessed [66–69,75,77,79,83,91,93,94,98,100]. Any definition of “sexual partner” was accepted for this outcome. Analyses represent at least 7,495 but fewer than 10,513 participants; one large trial restricted analyses to sexually experienced participants without reporting the size of this subgroup [100]. Four evaluations found significantly protective effects at any follow-up compared to attention controls (*n* = 665) [77,98], usual care (*n* = 1,412) [68], or a nonenhanced program version (*n* = 159) [94]. The remaining nine trials found no significant effects (3,842 < *n* < 6,860) [66,67,69,75,79,83,91,93,100].

Two school-based trials (*n* = 1,842) reported the number of partners with whom participants reported unprotected sex [67,69]. One trial found significantly protective effects among all participants at 19 mo and 31 mo follow-up compared to an information control (*n* = 1,371) [67]; repeated measures analyses by gender found significance among males only (*n* = 658). The other trial found no significant effects at 6, 12, or 18 mo follow-up compared to usual care (*n* = 471) [69].

#### Condom use.

In 26 trials a measure of condom use was reported [62–69,71,76–78,80–83,87,89,91,93–95,97–100]. Analyses represent at least 5,100, but fewer than 14,641 participants; nine trials did not report the exact number of participants analyzed [65,68,71,76,80,91,93,94,100]. A significantly protective effect was reported by 14 trials at any follow-up compared to no treatment (*n* < 500) [71], attention controls (378 < *n* < 2,085) [77,78,80,98], information about HIV (*n* = 1,436) [62,63,67], usual care (*n* = 515) [66,69], or a nonenhanced program version (*n* < 695) [64,91,94,95]. The remaining 12 trials found no significant effects (2,432 < *n* < 9,382) [65,68,76,81–83,87,89,93,97,99,100]. No trial assessed the use of male and female condoms separately.

An additional trial assessed the absolute number of times that all participants used condoms at 6 wk follow-up, finding no significant effects in an ANOVA comparing abstinence-plus, abstinence-only, and safer-sex programs against a no-treatment control (*n* = 388) [75]. A post-hoc test found that safer-sex program participants reported using condoms on significantly more occasions than abstinence-plus program participants (*n* ≈ 194). However, the trial did not relate the data to the number of times participants had sexual intercourse, making it impossible to say whether this effect suggests benefit (e.g., fewer sex acts) or harm (e.g., a lower percentage of condom-protected occasions).

#### Sexual initiation.

Sexual initiation, virginity, or “ever had sex” was assessed in 19 trials (*n* ≈ 20,367) [62,63,65,67–70,73,74,83–89,97,99,100]. In four a significantly protective program effect was found at any time point compared to usual care (*n* = 1,683) [65,68], or a nonenhanced program version (*n* = 277) [74,88]. The other 15 trials found no significant effects (*n* ≈ 16,728) [62,63,67,69,70,73,83–87,89,97,99,100].

### Knowledge, Cost, and Acceptability

A measure of HIV/AIDS knowledge was reported by 24 trials (*n* ≈ 20,904) [62,66–72,74,76–79,81–83,91–95,97,99,100]. In 20 trials (*n* ≈ 19,364) it was observed that abstinence-plus program participants reported significantly greater HIV/AIDS knowledge when compared to various controls [62,66–72,74,76–79,81–83,91,93,99,100]. In one trial, participants in a nonenhanced program version (without peer counselling) demonstrated greater knowledge than participants in the enhanced version at 3 mo follow-up (*n* = 52) [74]. The four trials with nonsignificant findings compared an abstinence-plus program to a nonenhanced program version (*n* = 494) [92,94,95], or used an attention control in an area with pre-existing HIV education (*n* = 938) [97].

Insufficient cost data were available to assess the overall cost-benefit of abstinence-plus interventions. Where information about program acceptability was reported (23 trials), evaluations consistently indicated high levels of acceptability and participant satisfaction [62,67–69,73,76–79,84–95,98,100].

## Discussion

The 39 included trials (baseline *n* ≈ 37,724) showed no evidence that abstinence-plus programs increase HIV risk among youth participants in high-income countries, and multiple evaluations found that the programs can decrease HIV risk. In 24 trials (baseline *n* = 20,982) significantly protective program effects were observed for behavioural [62–69,71,73,74,77–80,84,85,88,90,91,94,95,98] or biological [64,89] outcomes.

This review found no conclusive evidence that abstinence-plus programs can affect STI incidence and found limited evidence suggesting that abstinence-plus programs can reduce pregnancy incidence; however, the direction of findings consistently favoured abstinence-plus programs over any controls. Programs had mixed effects on sexual behaviour: individual trials discovered protective effects on incidence and frequency of unprotected vaginal, anal, and oral sex; incidence and frequency of vaginal and anal sex; incidence of any sexual activity; number of partners; number of unprotected partners; condom use; and sexual initiation. The trials that assessed HIV/AIDS knowledge found significant results favouring the majority of abstinence-plus program participants over various controls. No adverse effects were reported for any outcome.

### Additional Results from Evaluations That Could Not Be Obtained

At the time of this review, two replication trials of Be Proud! Be Responsible! had been completed, but we were unable to obtain complete results from conference presentations or the authors in time for this review [140,141]. We do not believe that including these trials would have changed our findings.

One unpublished trial encompassed ten arms (baseline *n* = 662), comparing an 8 h and a 12 h abstinence-plus program to an abstinence-only program, a safer-sex program, and an attention control at 24 mo follow-up (with and without booster sessions for each condition) [141]. Preliminary findings showed no significant differences in sexual initiation among baseline virgins between the abstinence-plus and the abstinence-only arms. Similar analyses of sexual intercourse incidence among all participants appeared to favour the abstinence-only intervention over the abstinence-plus intervention arms, although significance was marginal (*n* < 336, *p* = 0.05). Outcome data for all other comparisons, including results for condom use and unprotected sex, were unavailable. This trial is classified as “ongoing” in Figure 1, as it was recently completed.

The other trial took place among slightly older adolescents enrolled in ten US suburban high schools [140]. Preliminary findings suggested no significant behavioural effects at 1 y follow-up when compared to an attention control [33], but specific results were unavailable. This trial is classified as “full text unavailable” in Figure 1.

### Study Strengths

This is the first review to our knowledge to focus exclusively on abstinence-plus programs for HIV prevention among any participants in high-income countries. Our review adds to previous assessments of abstinence-plus programs by virtue of its international scope; prespecified, systematic, and highly sensitive search for trial evidence; inclusion of published and unpublished literature; extensive scrutiny of methodology of included trials; exclusive focus on trials reporting behavioural and biological outcomes; prereviewed protocol [41]; independence from external funding; assessment of data on cost, participant satisfaction, and program implementation; and acceptance of only the most rigorous trial evidence (i.e., data from randomized and quasi-randomized controlled trials).

This also is the first review to our knowledge to extensively search for abstinence-plus program trials from all high-income countries. Our findings suggest that abstinence-plus program trials outside North America are rare or inaccessible by existing search methods. We concur with past reviews indicating that abstinence-based approaches are less common outside the US [142,143]. Some abstinence-plus programs may be feasible beyond North America; however, program implementers must investigate program acceptability and rigorously compare abstinence-plus programs to existing HIV prevention strategies.

### Generalisability and Study Limitations

These results may not generalize to all abstinence-plus programs in all high-income settings. The included trials most frequently evaluated community-based abstinence-plus programs among US ethnic minority adolescents from low-income urban areas. However, application of this trial evidence in any high-income setting must be considered carefully given heterogeneity and the study limitations outlined above.

It was not possible to carry out subgroup analyses by participant or study characteristics, although many of these may be important confounders of program effects. Age may be a particularly critical moderator: with mean ages ranging from 11.3 to 19.3 y, trials enrolled participants in many stages of sexual development, which might influence the way abstinence-based messages were presented and received across trials. It was also difficult to conduct subgroup analyses based on different program definitions of abstinence, as most trials did not specify an exact definition.

Despite our extensive search for unpublished and ongoing trials, publication bias and missed trials are always concerns for systematic reviews. This review does not include studies indexed after February 2007. We did not use a Bonferroni or other correction to control for multiple statistical tests. The results reported in this paper summarize 562 separate statistical tests, of which 155 attained statistical significance at a level of *p* < 0.05 (far more than the 28 tests that may have been expected to attain significance by chance).

Perhaps owing to software limitations and lack of access to the original data sets, our reanalyzed results differed slightly from originally published results in trials by Boekeloo et al. (incidence of vaginal sex [66]), Dancy et al. (sexual initiation [70]), Jemmott et al. (incidence [78] and frequency [78,79] of unprotected vaginal sex), Moberg et al. (sexual initiation [87]), Sikkema et al. (condom use and sexual initiation [63]), St Lawrence et al. (incidence of casual sex, knowledge [92]), Villarruel et al. (incidence of vaginal sex [98]), and Wu et al. (pregnancy and incidence of vaginal sex [64]). All but three differences were in the direction of nonsignificant effects in our reanalyzed version; reanalyzed results were significant for incidence of unprotected vaginal sex in one trial [78], condom use in a second trial [63], and pregnancy in a third trial [64].

Methodological characteristics of the primary trials may further affect the generalisability of our conclusions. Strengths across trials included using relatively large sample sizes, reporting long-term follow-up data, stating a theoretical basis for the experimental intervention, describing the development of data collection instruments, using techniques to promote the validity of self-reported data, controlling for baseline differences, and reporting the causes and possible impacts of attrition. Deficiencies included the underreporting of key methodological and statistical information, attrition exceeding 33% in eight trials [63,64,68,69,81,82,99,100], lack of controls for clustered randomization, self-reported outcomes, vulnerability to floor effects, and insufficient reporting on program design and implementation. These deficiencies mirrored limitations we observed in our review of abstinence-only program trials [144].

### Clinical Relevance

Despite these limitations, the evidence from this systematic review has crucial implications for public health policy and practice, particularly in the debate over abstinence-only and abstinence-plus HIV prevention strategies. Our review of abstinence-only programs [43,44] discovered 13 program trials from eight papers [75,145–151], which enrolled 15,940 US adolescents. Trials consistently found no significant program effects on most biological and behavioural outcomes when compared to various controls; isolated findings of benefit and harm were offset by nonsignificant findings in other trials. In sum, the review suggested that abstinence-only interventions do not significantly decrease or exacerbate HIV risk among high-income country participants.

In contrast, this review suggests that numerous abstinence-plus interventions can have significantly protective effects on multiple sexual risk behaviours when compared to various controls; furthermore, abstinence-plus programs did not have adverse effects on behavioural or biological outcomes. Participants appeared to understand the hierarchical message of abstinence-plus programs, as trials consistently reported significant program effects for HIV prevention knowledge. No trial observed adverse effects on incidence or frequency of sexual activity, suggesting that safer sex promotion did not encourage sex. Moreover, the promotion of abstinence did not appear to detract from the programs' condom promotion message: many trials found protective short-term and long-term effects on condom use, and no trial found an adverse effect.

Given that HIV risk in the US is elevated among low-income and ethnic minority populations, we originally planned to conduct subgroup analyses based on these characteristics [41]. The lack of a quantitative synthesis made these comparisons difficult. However, protective behavioural and biological program effects were frequently observed among the 29 trials that enrolled primarily ethnic minority participants [62–70,73,74,76–84,88–91,93,95,96,98,99], the 12 trials that enrolled primarily African-American participants [62,64,70,73,74,76–79,91,95,96], and the 18 trials that reported enrolling economically disadvantaged participants [62–65,70,73,74,77–79,84,88–91,95,96,99]. Although these findings cannot provide definitive conclusions about the moderating effects of ethnicity or socioeconomic status, they suggest that some abstinence-plus programs may be appropriate, acceptable, and effective for underserved youth populations.

### Future Research

While many trials in this review observed protective behavioural or biological effects, more research is necessary to understand what contexts, populations, and program elements make these effects possible. Only one trial directly compared an abstinence-plus against an abstinence-only or a safer-sex intervention [75], and more comparisons of this type may be necessary. Faith-based programs were not represented, suggesting that they have not yet been evaluated using rigorous methodology. Several evaluations found that one version of an abstinence-plus program was more effective than another, prompting research into intervention mechanisms. Additional trials might also clarify program effects and acceptability among non-North American youth and other underrepresented groups (e.g., nonheterosexual youth, youth with disabilities, or recent immigrants).

In future primary trials, key methodological, clinical, and statistical information should be reported more completely (i.e., following the CONSORT [Consolidated Standards of Reporting Trials] statement [152]). To help policymakers and practitioners, it is also necessary to report implementation information fully for intervention and control arms. Clearer reporting could help identify precisely which program components were effective across trials. Data analyses should account for dropouts, clustered randomization, and multiple statistical tests, and data sets and intraclass correlation coefficients should be provided.

Finally, inconsistency in outcome measures across studies suggests that trialists lack a standardized set of outcome measures relevant to HIV risk. Consensus among HIV prevention trialists is necessary to define relevant outcomes with consistent recall periods and clinical meanings; this will assist future research syntheses. Oral, anal, and vaginal sex acts carry different HIV-related risks [153–155] and should be evaluated separately. Same-sex sexual behaviours should also be assessed. Medical assessments of STI and HIV incidence are vital for understanding HIV risk, but these have been severely underutilized to date. Even relatively small trials should attempt to use biological end points, as the aggregation of small trials in a quantitative synthesis could overcome the problem of floor effects. Recent studies in high-income countries suggest that school-based STI screening is acceptable among general adolescent populations [156,157], encouraging future efforts to evaluate biological end points among young people.

## Supporting Information

Table S1 Participant and Intervention Characteristics CBT, cognitive-behavioural theory; HBM, health belief model; IMB, information-motivation-behavioural skills theory; SCT, social cognitive theory; SES, socioeconomic status; SLT, social learning theory; TPB, theory of planned behaviour; TRA, theory of reasoned action.
(105 KB DOC)

Text S1 List of High-Income Economies (24 KB DOC)

Text S2 PubMed Search Strategy (25 KB DOC)

Text S3 QUOROM Checklist (45 KB DOC)

## Abbreviations

ACASI: audio computer-assisted self interviewing
STI: sexually transmitted infection
WHO: World Health Organization
