# Supplementary material for: Systematic Review of Abstinence-Plus HIV Prevention Programs in High-Income Countries
Source: PLoS Med. 2007 Sep 18;4(9):e275. doi: 10.1371/journal.pmed.0040275 (PMC1976624; doi:10.1371/journal.pmed.0040275)
Supplement: Supplementary file 1 [file pmed.0040275.sd001.doc]

**High-Income Economies** [45]

Andorra, Antigua and Barbuda, Aruba, Australia, Austria, the Bahamas, Bahrain, Belgium, Bermuda, Brunei Darussalam, Canada, Cayman Islands, Channel Islands, Cyprus, Denmark, Faeroe Islands, Finland, France, French Polynesia, Germany, Greece, Greenland, Guam, Hong Kong (China), Iceland, Ireland, Isle of Man, Israel, Italy, Japan, Korea Rep., Kuwait, Liechtenstein, Luxembourg, Macao (China), Malta, Monaco, Netherlands, Netherlands Antilles, New Caledonia, New Zealand, Norway, Portugal, Puerto Rico, Qatar, San Marino, Saudi Arabia, Singapore, Slovenia, Spain, Sweden, Switzerland, United Arab Emirates, United Kingdom, United States, US Virgin Islands
