# Supplementary material for: Systematic Review of Abstinence-Plus HIV Prevention Programs in High-Income Countries
Source: PLoS Med. 2007 Sep 18;4(9):e275. doi: 10.1371/journal.pmed.0040275 (PMC1976624; doi:10.1371/journal.pmed.0040275)
Supplement: Supplementary file 2 [file pmed.0040275.sd002.doc]

**PubMed Search Strategy**

[#1](javascript:PopUpMenu2_Set(QMenu1);)	Search HIV Infections[MeSH] OR HIV[MeSH] OR hiv[tw] OR hiv-1*[tw] OR hiv-2*[tw] OR hiv1[tw] OR hiv2[tw] OR hiv infect*[tw] OR human immunodeficiency virus[tw] OR human immunedeficiency virus[tw] OR human immuno-deficiency virus[tw] OR human immune-deficiency virus[tw] OR ((human immun*) AND (deficiency virus[tw])) OR acquired immunodeficiency syndrome[tw] OR acquired immunedeficiency syndrome[tw] OR acquired immuno-deficiency syndrome[tw] OR acquired immune-deficiency syndrome[tw] OR ((acquired immun*) AND (deficiency syndrome[tw])) OR "Sexually Transmitted Diseases, Viral"[MeSH:NoExp] Limits: Publication Date from 1980 to 2007

[#2](javascript:PopUpMenu2_Set(QMenu2);)	Search Sexual Abstinence [MeSH] Limits: Publication Date from 1980 to 2007

[#3](javascript:PopUpMenu2_Set(QMenu3);)	Search abstinence OR abstain* OR chastity OR chaste OR virgin* OR celiba* OR (sex* AND educat*) OR (sex* AND (marriage* OR married)) Limits: Publication Date from 1980 to 2007

[#4](javascript:PopUpMenu2_Set(QMenu4);)	Search #2 OR #3 Limits: Publication Date from 1980 to 2007

[#5](javascript:PopUpMenu2_Set(QMenu5);)	Search randomized controlled trial [pt] OR controlled clinical trial [pt] OR randomized controlled trials [mh] OR random allocation [mh] OR double-blind method [mh] OR single-blind method [mh] OR clinical trial [pt] OR clinical trials [mh] OR ("clinical trial" [tw]) OR ((singl* [tw] OR doubl* [tw] OR trebl* [tw] OR tripl* [tw]) AND (mask* [tw] OR blind* [tw])) OR ( placebos [mh] OR placebo* [tw] OR random* [tw] OR research design [mh:noexp] OR comparative study [mh] OR evaluation studies [mh] OR follow-up studies [mh] OR prospective studies [mh] OR control* [tw] OR prospectiv* [tw] OR volunteer* [tw]) NOT (animals [mh] NOT human [mh]) Limits: Publication Date from 1980 to 2007

[#6](javascript:PopUpMenu2_Set(QMenu6);)	Search #1 AND #4 AND #5 Limits: Publication Date from 1980 to 2007
