# Supplementary material for: Systematic Review of Abstinence-Plus HIV Prevention Programs in High-Income Countries
Source: PLoS Med. 2007 Sep 18;4(9):e275. doi: 10.1371/journal.pmed.0040275 (PMC1976624; doi:10.1371/journal.pmed.0040275)
Supplement: Supplementary file 4 [file pmed.0040275.st001.doc]

TABLE S1. PARTICIPANT AND INTERVENTION CHARACTERISTICS

| **Study** | **Setting** | **Participants** | **Intervention** | **Control** | **Last follow-up, attrition** |
| --- | --- | --- | --- | --- | --- |
| Aarons 2000 [65] | Middle schools; Washington, DC | 582 students, mean age=12.8, 48% male, primarily minority, SES low | 7.5 hours over 2 years, SCT, classroom delivery by peer and adult leaders, no parent involvement. | Usual care defined by schools | 15 months, 28% |
| Boekeloo 1999 [66] | Pediatrician offices; Washington, DC | 219 primary care patients, mean age=13.5, 50% male, primarily minority, SES unclear | 0.5 hours in 1 session, SCT/TRA, one-on-one delivery by pediatrician, parent brochures. | Usual care pediatrician contact | 9 months, 10% |
| Coyle 2001 [67] | High schools; sites in CA & TX | 3869 students, mean age=14, 50% male (intervention) 46% male (controls), primarily minority, SES diverse | 20 hours over 2 years, SCT/social influences, classroom delivery by peer and adult leaders, parent newsletters and parent-child homework. | Information. 5 class sessions focusing on HIV/STD knowledge. Unclear theory, parent involvement, leaders | 31 months, 21% |
| Coyle 2004 [68] | Middle schools; sites in CA | 2829 students, mean age=11.5, 50% male, primarily minority, SES diverse | 20 hours over 3 years, SCT/social inoculation, classroom delivery by adult leaders, no parent involvement. | Usual care defined by schools | 36 months, 36% |
| Coyle 2006 [69] | Alternative schools; northern CA | 988 students, mean age=16.0, 62% male, primarily minority, SES unclear | 26 hours over 6 weeks: 13.5 hours classroom delivery by health educators, 12.5 hrs community service as a classroom, SCT/TRA/TBP, no parent involvement. | Usual care defined by schools | 18 months, 58% |
| Dancy 2006 [70] | Communities with high poverty levels; Chicago, IL | 262 girls, mean age=12.4, 0% male, 100% African-American, SES low | #1. Mother-led. 12 prep weeks of training for mothers led by health experts, then 12 hours small-group sessions for girls over 6 weeks led by mothers. SCT/TRA/community-other mothers.  #2. Health educator-led. 12 hours small-group sessions for girls over 6 weeks led by health experts. | Attention. Same format as #1, but focused on nutrition and exercise (not HIV). | 5.5 months, 11% |
| Danella 2000 [71] | Schools and STD clinic; Nassau, Bahamas | 500 girls, aged 9-17, 0% male, Bahamian ethnicity, SES unclear | 15 hours over 10 weeks with graduation ceremony. SCT/PMT, information and skills delivered by adults in group settings. Booster session after 6 months. Lectures, videos, games, interactive activities. Addressed alcohol, sexual harassment, abuse, “sweethearting,” HIV. Unclear if parents involved. | No treatment (delayed receipt of the experimental program). | 12 months, unclear attrition |
| Danielson 1990 [72] | Medical exam rooms; sites in OR & WA | 1195 males in primary care, mean age=16.5, 100% male, primarily white, SES unclear | 1 hour in 1 session, theory not stated, one-on-one delivery by health professional, no parent involvement. | No treatment | 12 months, 19% |
| DiIorio 2006 [62] | Community-based youth programs; Atlanta, GA | 582 youth, mean age=12.2, 60% male, 97.9% African-American, SES low | #1. SCT. 14 hours over 14 weeks for mothers and adolescents, led by adult facilitators. Focus on adolescent development and communication.  #2. Life skills. 14 hours over 14 weeks for mothers and adolescents, led by adult facilitators. Problem behavior theory. Focus on many at-risk behaviors and community involvement. | Information. 1-hr session for mothers and adolescents including a videotape and discussion about HIV prevention. | 24 months, 10% |
| DiIorio 2007 [73] | Community-based youth programs; Atlanta, GA | 277 boys, mean age=12.7, 100% male, 97% African-American, SES low | 14 hours over 7 weeks: 6 sessions for fathers and 1 session for fathers and sons, in small groups led by adult facilitators. SCT. Information provision and communication skills. | Attention. Same format as experimental, but focused on nutrition and exercise. | 12 months, ≈20% |
| Ferguson 1998 [74] | After-school program; Charlottesville, VA | 63 after-school program participants, mean age=13.3, 0% male, 100% African-American, SES low | 16 hours over 8 weeks, theory not stated, classroom delivery by adult with Africentric counseling by peer counselors, no parent involvement. | Non-enhanced. Same as experimental, but without peer counseling component. All activities adult-led. | 3 months, 17% |
| Hernandez 1990 [75] | University lecture rooms; NC | 410 university students, mean age=19.3, 55% male, primarily white, SES unclear | 0.75 hours in 1 session, theory not stated, classroom delivery via video and written materials, no parent involvement. Prioritized abstinence over condom use, but encouraged both. | #1. Abstinence-only. Same as experimental, but did not mention or promote condom use.  #2. Safer-sex. Same as experimental, but did not prioritize abstinence over condom use.  #3. No treatment | 1.5 months, 5% |
| Hewitt 1998 [76] | Middle school (on Saturday); Philadelphia, PA | 124 youth recruited from schools, mean age=13.2, 39% male, 100% African-American, SES unclear | #1. Africentric. 5 hours in 1 session, SCT/TPB, small group delivery by adult leader, no parent involvement. Integrated aspects of African-American culture and pride.  #2. Generic. Same as above, but did not integrate African-American culture. | Attention. Same format as experimental, but focused on general health. No HIV prevention component. | 3 months, 4% |
| Jemmott 1992 [77] | Middle school (on Saturday); Philadelphia, PA | 157 boys recruited from schools, mean age=14.6, 100% male, 100% African-American, SES low | 5 hours in 1 session, SCT/TRA/TPB, small group delivery by adult leaders, no parent involvement. | Attention. Same format as experimental, but focused on career planning. No HIV prevention component. | 3 months, 4% |
| Jemmott 1998 [78] | Middle school (on Saturday); Philadelphia, PA | 659 youth recruited from schools, mean age=11.8, 47% male, 100% African-American, SES low | #1. Abstinence-focused. 8 hours over 2 weeks, SCT/TRA/TPB, small group delivery by peer or adult leaders, no parent involvement. Abstinence emphasized, but stated that condoms can also prevent HIV.  #2. Condom-focused. Same, with conceptual difference. Condom use emphasized, but stated that abstinence is the best prevention strategy. | Attention. Same format as experimental, but focused on general health promotion. No HIV prevention component. | 12 months, 7% |
| Jemmott 1999 [79] | Middle school (on Saturday); Trenton, NJ | 496 youth, mean age=13.2, 46% male, 100% African-American, SES low | 5 hours in 1 session, SCT/TRA/TPB, small group delivery by adult leaders, no parent involvement. | Attention. Same format as experimental, but focused on general health promotion. No HIV prevention component. | 6 months, 7% |
| Jemmott 2004 [80] | Community-based organization; PA, NJ | 3448 youth randomized, 1707 randomly selected for assessment, mean age=14.8, 43% male, primarily minority, SES unclear | 8 hours over 2 weeks, SCT/TRA/TPB, small group delivery by adult leaders, no parent involvement. | Attention. Same format as experimental, but focused on general health promotion. No HIV prevention component. | 12 months, ≥9% |
| Kennedy 2000a [81] | Community center; Sacramento, CA | 735 youth, mean age=15.3, 45% male, primarily minority, SES unclear | 5-9 hours in 1-2 sessions, TRA, small group delivery by peer leaders, no parent involvement. | No treatment | 1 month, 48% |
| Kennedy 2000b [82] | Community center; Nashville, TN | 535 youth, mean age=14.4, 41% male, primarily minority, SES unclear | 5-9 hours in 1-2 sessions, TRA, small group delivery by adult leaders, no parent involvement. | No treatment | 1 month, 44% |
| Kirby 1997 [83] | Middle schools; Los Angeles, CA | 2099 students, mean age=12.3, 46% male, primarily minority, SES unclear | 8 hours over 2 weeks, SLT/HBM, classroom delivery by peer leaders who have experienced teen pregnancy or HIV, no parent involvement. | Usual care defined by schools | 17 months, 23% |
| Markham 2006 [84] | Middle schools; sites in TX | 1307 students, mean age=12.5, 43% male, primarily minority, SES low | 22 hours over 2 years: 12 sessions in 7^th^ grade and 10 in 8^th^ grade. SCT. Classroom delivery by adults, 4 individual computer-based lessons, parent-child homework. | Usual care defined by schools. | 5 months, 8% |
| McBride 2000a [85] | Middle and high schools; sites in WA | 166 at-risk girls, mean age=14.9, 0% male, primarily white, SES unclear | Mean 31 hours over 8 months, client-centered model, small group delivery by adult leaders, no parent involvement. | No treatment | 8 months, 20% |
| McBride 2000b [86] | Schools and community centers; sites in WA | 232 at-risk girls, mean age=15.4, 0% male, primarily white, SES unclear | Mean 22 hours over 6 months, client-centered model, small group delivery by adult leaders, no parent involvement. | No treatment | 6 months, 32% |
| Moberg 1998 [87] | Middle schools; sites in WI | 2483 students, mean age=14, 48% male, primarily white, SES unclear | #1. Age-appropriate. 54 hours delivered over 3 years (grades 6-8), social influences theory, classroom delivery by adults, parent meetings & mailings, parent-child homework.  #2. Intensive. Same as above, but all 54 hours delivered in 1 year (grade 7). | Usual care defined by schools. | 48 months, 32% |
| O'Donnell 2002 [88] | Middle schools; Brooklyn, NY | 225 students, mean age=12.4, 41% male, primarily minority, SES low | 254 hours over 2 years. 40 in-school classes in 7^th^ grade and 34 classes in 8^th^ grade delivered by adults, plus 90 hours of after-school community service per year. Theory not stated, parents helped design curriculum. | Non-enhanced. Same in-school curriculum as experimental arm, but without any community service hours. | 44 months, 24% |
| Philliber 2001 [89] | Community centers; sites in NY, MD, TX, FL, OR, WA | 1163 youth, mean age=14.3, 45% male, primarily minority, SES low | 2250 hours offered over 3 years (mean attendance = 432 hours over 3 years). Parallel family systems, one-on-one and group delivery by adults, parent orientation session. | Usual care defined by community centers. | 36 months, 19% |
| Sikkema 2005 [63] | Low-income housing developments, sites in VA, WI, WA | 1172 youth, mean age=14.5, 50% male, primarily minority, SES low | #1. Workshop. 6 hours in 2 sessions delivered by adults (over 2 weeks), in separate groups by gender. Brochures, free condoms, skills training. SCT/TRA/TPB/social learning. No parent involvement.  #2. Community-level. Same as above, with 2 follow-up sessions, (peer) teen leadership council, teen-council-sponsored activities, and parent workshop, over 12 months. | Information. Participants viewed and discussed an HIV prevention video. Brochures and free condoms were distributed. 15% of participants attended. | 18 months, 35% |
| Smith 1994 [90] | High school; New York, NY | 120 students, mean age=15.1, 26% male, primarily minority, SES low | 8 hours of classroom sessions delivered by adult, 6-week career-based mentorship placement, additional classroom sessions with unclear dosage. Theory not stated, no parent involvement. | Information. Participants were given written materials on contraception and sexual decision making. | 6 months, 21% |
| St Lawrence 1995a [91] | Community health centers; Jackson, MS | 246 youth, mean age=15.3%, 28% male, 100% African-American, SES low | 15 hours over 8 weeks, social learning theory, classroom delivery by adults, no parent involvement. Information and behavioral skills training. | Non-enhanced. Same as experimental, but provided information only, without behavioral skills training. Small-group delivery by adults in one 2-hour session. | 12 months, 9% |
| St Lawrence 1995b [92] | Residential drug treatment center; Jackson, MS | 34 youth in treatment for substance abuse, mean age=15.6, 73% male, 16% minority, SES unclear | 9 hours over 6 weeks, CBT, small group delivery by adults, no parent involvement. Information and behavioral skills training. | Non-enhanced. Same as experimental, but without skills training. | 2 months, 0% |
| St Lawrence 1999 [93] | Juvenile reformatory, MS | 361 male juvenile offenders entering a correction facility, mean age=15.8, 100% male, 72% minority, SES unclear | 6 hours over 3 weeks, CBT, small group delivery by adults, no parent involvement. Information and behavioral skills training. | Attention. Same format as experimental, but focused on anger management skills. | >7 months, 14% |
| St Lawrence 2002 [94] | Residential drug treatment center; MS | 161 youth in treatment for substance abuse, mean age=16.0, 68% male, 25% minority, SES unclear | #1. 18 hours over 4 weeks, IMB, small group delivery by adults, no parent involvement. Information, behavioral skills training, and “motivation” component in last session (distributed photographs altered to show participants in end-stage AIDS).  #2. Same as above, but without distributing motivational photographs to personalize AIDS risk. | Non-enhanced. 18 hours over 4 weeks, small group delivery by adults, no parent involvement. First session (1-1.5 hours) focused on HIV prevention and advised abstinence and condom use. Subsequent sessions focused on general health, gangs, weapons, stress. | 13.5 months, 11% |
| Stanton 1996 [95] | Public housing developments, Baltimore, MD | 383 youth, median age=11.3, 56% male, 100% African-American, SES low | 10.5 hours over 7 weekly sessions plus one day-long session at a campsite. SCT/PMT, delivery by adults to natural single-sex friendship groups. Cultural components and decision-making, condoms provided. Parents on advisory board. | Non-enhanced. Health education program: videos and discussion of various health topics, condoms provided. No cultural components, decision-making, or focus on friendship. | 12 months, 27% |
| Stanton 2000 [96] | Public housing developments, Baltimore, MD | 237 youth, median age=13.6, 51% male, 100% African-American, SES low | 1.25 hours in 1 session delivered by adults to parent-youth dyads in the family home. SCT, focused on parental monitoring for risk behaviors, included a video, roleplays and condom skills practice. | Attention. Same format as experimental, but focused on career goals instead of HIV prevention. | 12 months, 24% |
| Stanton 2006 [97] | Schools and community organizations; 12 rural WV counties | 1131 youth, mean age≈14.3, 42% male, primarily white, SES unclear | #1. Standard. 10.5 hours over 1-2 days. SCT/PMT, delivery by adults to natural single-sex friendship groups. Included games, discussion, homework, and videos. Prelim focus groups included parents. Usually no skills practice with condoms.  #2. Culturally Adapted. Same as 1, but culturally adapted for WV rural youth, more factual info, exercises on goal setting and planning. 132 youth received the program via interactive television. Grouped with 1 for analysis. | Attention. Group-based curriculum about environmental education. No focus on decision-making, abstinence, HIV, drugs, alcohol, or drug-selling. | 9 months, 20% |
| Villarruel 2006 [98] | High schools (on Saturday); Philadelphia, PA | 656 eligible and ineligible youth randomized. Among 553 eligible youth: mean age=14.9, 45% male, 100% Latino, SES unclear | 8 hours over 2 weeks, SCT/TRA/TPB, small group delivery by adult leaders, no parent involvement. Incorporated elements of Latino culture. | Attention. Same format as experimental, but focused on general health promotion. No HIV prevention component. | 12 months, 18% of randomized youth |
| Weeks 1997 [99] | Middle schools; Chicago, IL | 2392 students, mean age=12.5, 51.4% male, primarily minority, SES low | #1. 15 hours over 2 years, SCT/social influences, classroom delivery by adults, parent meetings, parent-child homework.  #2. Same as above, but less parent involvement. Parent meetings were held, but no parent-child homework. | Usual care defined by school district. “Basic, minimal AIDS education in compliance with state mandates.” | 24 months, 55% |
| Wright 1997 [100] | High schools; sites in Manitoba, New Brunswick, Nova Scotia, and Ontario, Canada | 4512 students, mean age=14.5, 42% male, ethnicity unclear, SES unclear | 20 hours over 1 academic year, IMB/TRA/TPB/self-efficacy theory, classroom delivery by adults and peer leaders, parent-child activities included. | Usual care defined by schools. Usually focused on didactic instruction, average exposure was <20 hours. | 24 months, 49% |
| Wu 2003 [64] | Housing developments and community centers; Baltimore, MD | 817 youth, median age=14, 42% male, 100% African-American, SES low | #1. FOK and ImPACT: 10.5 hours over 7 weekly sessions plus one day-long session at a campsite. SCT/PMT, delivery by adults to natural single-sex friendship groups. 1-session parent-child monitoring intervention delivered by trial staff in each home, including a video, roleplays and condom demonstration.  #2. FOK, ImPACT, and boosters. Same as #1, with group booster sessions for youth at 6 and 10 months. Grouped with #1 for analysis, no difference between #1 and #2 in sub-analyses. | Non-enhanced. FOK delivery same as #1, but the added parent-child monitoring (ImPACT) intervention focused on career goals instead of HIV prevention. | 24 months, 40% |

CBT = cognitive-behavioral theory. HBM = health belief model. IMB = information-motivation-behavioral skills theory. SCT = social cognitive theory. SES = socio-economic status. SLT = social learning theory. TPB = theory of planned behavior. TRA = theory of reasoned action.
